# Resting State Networks and Consciousness: Alterations of Multiple Resting State Network Connectivity in Physiological, Pharmacological, and Pathological Consciousness States

**DOI:** 10.3389/fpsyg.2012.00295

**Published:** 2012-08-27

**Authors:** Lizette Heine, Andrea Soddu, Francisco Gómez, Audrey Vanhaudenhuyse, Luaba Tshibanda, Marie Thonnard, Vanessa Charland-Verville, Murielle Kirsch, Steven Laureys, Athena Demertzi

**Affiliations:** ^1^Coma Science Group, Cyclotron Research Center & Neurology Department, University of LiègeLiège, Belgium; ^2^Radiology Department, CHU Sart Tilman HospitalLiège, Belgium; ^3^Anesthesiology Department, CHU Sart Tilman HospitalLiège, Belgium

**Keywords:** default mode network, resting state networks, consciousness, sleep, anesthesia, coma, hypnosis

## Abstract

In order to better understand the functional contribution of resting state activity to conscious cognition, we aimed to review increases and decreases in functional magnetic resonance imaging (fMRI) functional connectivity under physiological (sleep), pharmacological (anesthesia), and pathological altered states of consciousness, such as brain death, coma, vegetative state/unresponsive wakefulness syndrome, and minimally conscious state. The reviewed resting state networks were the DMN, left and right executive control, salience, sensorimotor, auditory, and visual networks. We highlight some methodological issues concerning resting state analyses in severely injured brains mainly in terms of hypothesis-driven seed-based correlation analysis and data-driven independent components analysis approaches. Finally, we attempt to contextualize our discussion within theoretical frameworks of conscious processes. We think that this “lesion” approach allows us to better determine the necessary conditions under which normal conscious cognition takes place. At the clinical level, we acknowledge the technical merits of the resting state paradigm. Indeed, fast and easy acquisitions are preferable to activation paradigms in clinical populations. Finally, we emphasize the need to validate the diagnostic and prognostic value of fMRI resting state measurements in non-communicating brain damaged patients.

## Introduction

**Figure 1:**
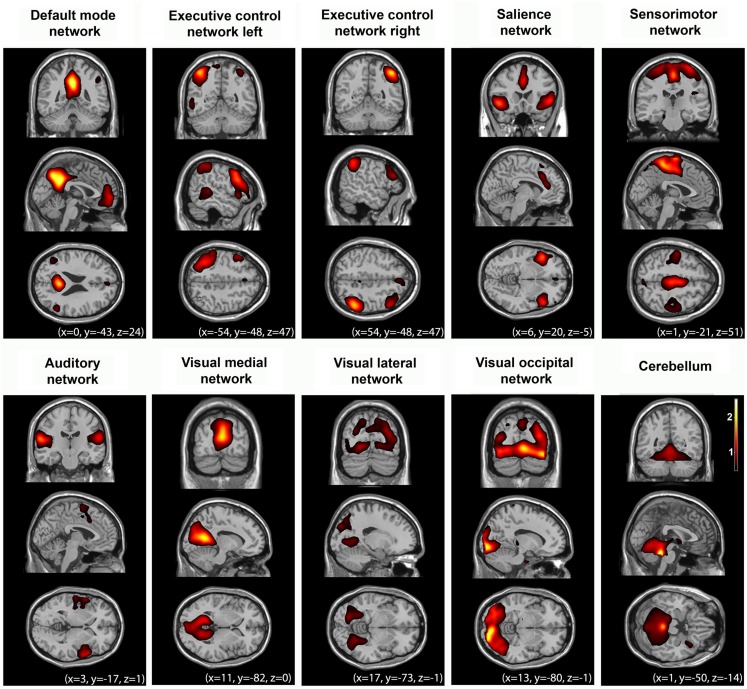
**Multiple cerebral networks can be identified with fMRI in healthy controls (*n* = 10) during normal wakeful resting state using independent component analysis**. These networks reflect “higher-order” cognitive (i.e., default mode, left and right executive control, salience networks), and “lower-order” sensorimotor, and sensory (auditory, visual) function. For illustrative purposes, group-level spatial maps (*z* values) are rendered on a structural T1 magnetic resonance template and *x*, *y*, and *z* values indicate the Montreal Neurological Institute coordinates of the represented sections.

In the past decades, neuroimaging research has been focusing on studying brain function in “resting” conditions, when subjects receive no external stimulation. Functional magnetic resonance imaging (fMRI) resting state connectivity studies stress that the brain at rest is characterized by coherent fluctuations in the blood-oxygen-level-dependent (BOLD) signal. These BOLD fluctuations can be detected in the low frequency range (<0.1 Hz; Cordes et al., 2001), they are distinct from respiratory and cardiovascular signal contribution (De Luca et al., 2006) and organize the brain in large-scale cerebral networks (Damoiseaux et al., 2006). The most widely studied resting state network (RSN) is the default mode network (DMN), encompassing precuneus/posterior cingulate cortex (PCC), mesiofrontal/anterior cingulate cortex (ACC), and temporoparietal junction areas (Figure 1). This network of areas was initially identified in positron emission tomography (PET) studies as regions less active when performance on cognitive tasks was compared to resting control condition, such as eye fixation or eyes closed (Shulman et al., 1997; Mazoyer et al., 2001). Later, the DMN was also identified in fMRI and in terms of cognitive function, its activity has been linked to self-related and internal processes, such as stimulus-independent thoughts (McKiernan et al., 2006), mind-wandering (Mason et al., 2007), social cognition (Schilbach et al., 2008), introspection (Goldberg et al., 2006), monitoring of the “mental self” (Lou et al., 2004), and integration of cognitive processes (Greicius et al., 2003). Interestingly, areas of the DMN can be assigned to specific cognitive functions, for example the PCC seems to be important in autobiographical memory while the frontal areas may be important for self-reference (Whitfield-Gabrieli et al., 2011). In that respect, resting state acquisitions can, at least to a certain degree, be informative of cognitive function. Importantly for clinical studies, the resting state paradigm is particularly appealing because it does not require sophisticated experimental setup to administer external stimuli and surpasses the need for patients’ contribution (e.g., language comprehension and/or production or motor responses; Soddu et al., 2011). Hence, resting state protocols are a suitable means to study clinical populations, in which communication cannot be established at the bedside, such as patients with disorders of consciousness [e.g., coma, “vegetative state (VS)”/unresponsive wakefulness syndrome (UWS), minimally conscious state (MCS)]. It has been suggested that resting state analyses can be used in a clinical setting to identify group differences, to obtain patient-specific diagnostic and prognostic information, to perform longitudinal studies and monitor treatment effects, to cluster heterogeneous diseases such as schizophrenia or even to guide treatments, such as surgical interventions (Fox and Greicius, 2010).

With an aim to better determine the functional role of resting state activity in healthy conditions and to further comprehend its contribution to clinical states, the present review will adopt a “lesion” approach. Indeed, patients’ neurological data can give us information about the functional role of the resting state activity to consciousness. We will review changes in functional connectivity in the DMN under physiological (sleep, hypnosis), pharmacological (sedation, anesthesia), and pathological (coma-related states) alteration of consciousness. The functional contribution of the anticorrelated activity between DMN and the “extrinsic” system to (un)conscious states will also be discussed. We will further focus on functional connectivity changes in multiple RSNs, such as the bilateral executive control, salience, sensorimotor, auditory, and visual networks (Beckmann et al., 2005; Damoiseaux et al., 2006; De Luca et al., 2006; Fox and Raichle, 2007; Smith et al., 2009). With regards to resting state assessments of severely brain-injured patients, we will highlight some methodological issues mainly in terms of hypothesis-driven seed-based correlation analysis and data-driven independent components analysis. Finally, we will attempt to contextualize our discussion within theoretical frameworks around conscious processes.

## (Un)Conscious States and Resting State Default Mode Network Activity

To date, there is no universal definition for consciousness covering all its essential characteristics (Zeman, 2001). Here, we define consciousness in an operational way based on clinical practice, stressing that consciousness can be reduced to two components, arousal and awareness (Posner et al., 2007). Arousal refers to the level of alertness and it is clinically evidenced by eyes opening. Awareness refers to the content of consciousness and it is clinically evidenced by command following or by observing non-reflex motor behavior, such as eye tracking and localized responses to pain (Posner et al., 2007). Sleep is the best example to describe the relationship between these two components: the drowsier we become as we move toward deep sleep, the less aware we get of our surroundings and ourselves (a notorious exception is the oneiric activity during rapid eye movement sleep during which we remain behaviorally unconscious; Hobson and Pace-Schott, 2002). Based on this definition, subjects in pathological and pharmacological coma (i.e., anesthesia) are not conscious because they are not awake (American Society of Anesthesiologists Task Force on Intraoperative Awareness, 2006). Similarly, under sedation (a drug-dose dependent impairment of consciousness) and hypnotic state (a suggestion-dependent alteration of conscious experience; The Executive Committee of the American Psychological Association – Division of Psychological Hypnosis, 1994) subjects report an altered state of awareness as they move toward lower wakefulness levels. A unique dissociation between arousal and awareness is observed in patients in a VS (also called UWS; Laureys et al., 2010) who recover wakefulness but their motor responses are merely reflexive and, hence, not indicative of conscious awareness (Laureys et al., 2005). Patients in VS/UWS should not be mistaken with patients in a MCS. Patients in MCS, although unable to functionally communicate with their environment, do show fluctuating remnants of willful behavior (Giacino et al., 2002). Based on the level of their purposeful behavioral repertoire, MCS patients were recently subcategorized as MCS+ (i.e., showing command following,) and MCS− (i.e., showing visual pursuit, localization of noxious stimulation, or non-contingent behaviors, such as appropriate smiling or crying to emotional stimuli; Bruno et al., 2011). This kind of clinical distinction highlights the importance of motor output to the evaluation of consciousness. Patients with a locked-in syndrome (LIS), however, have no means of producing speech, limb, or facial movements but still are awake and conscious (Posner et al., 2007). Evidently, by solely measuring motor responses, these patients can be mistaken for unconscious (Laureys et al., 2005). Similarly, consciousness in patients with aphasia can be underestimated if the clinician does not account for such deficit. As a consequence, valid motor- and language-independent assessment of residual brain function in non-communicating patients is of both clinical and ethical importance.

The resting state paradigm surpasses the requirement for motor output or language comprehension. To date, neuroimaging protocols investigating connectivity of the DMN during resting state are not conclusive as to its exact functional role. Nevertheless, resting state fMRI studies suggest that activity of this network is generally reduced as a function of the level of consciousness (Table 1). For example, it has been shown that with the advancement of sleep, connectivity between the frontal and posterior parts of the DMN decreases yet persists (Horovitz et al., 2009). Decreases in functional connectivity were also observed in PCC of the DMN under pharmacological unconsciousness with propofol (Boveroux et al., 2010; Schrouff et al., 2011) and sevoflurane (Martuzzi et al., 2010). Importantly for clinical populations, connectivity in the PCC was shown to be indistinguishable between controls and LIS patients, relatively preserved in MCS, significantly reduced in VS/UWS patients (Vanhaudenhuyse et al., 2010) and could not be identified in brain death (i.e., irreversible coma with absent brainstem reflexes; Boly et al., 2009). Similarly during a passive auditory task, DMN deactivations, which are thought to interrupt ongoing introspective processes, showed a reduction in MCS whereas VS/UWS patients did not show such task-induced deactivations (Crone et al., 2011). These studies suggest that DMN functional connectivity correlates, at least partially, with the level of ongoing conscious cognition. This is in agreement with functional connectivity studies on intermediate states of awareness. For example, in hypnotic state there is only relative (Demertzi et al., 2011b) or no connectivity decreases in the DMN (McGeown et al., 2009). Similarly, during moderate sedation, little (Greicius et al., 2008) or no changes (Stamatakis et al., 2010) in DMN connectivity have been observed. During light sleep there is no change (Horovitz et al., 2008; Larson-Prior et al., 2009). Nevertheless, in deep sleep brain activity shows increased modularity, which hinders the brain to integrate information and therefore might account for decreased consciousness during dreamless sleep (Boly et al., 2012).

**Table 1:** **FMRI studies showing alterations in resting state functional connectivity of multiple networks in physiological (sleep, hypnosis), pharmacological (sedation), and pathological states of unconsciousness**.

		*N*	Functional connectivity change	Method	Study
DMN	Light sleep	14	Connectivity persists	Seed-based	Horovitz et al. (2008)
		10	Connectivity persists	Seed-based	Larson-Prior et al. (2009)
	Slow wave sleep	14	↑: PCC correlation with IPC. Correlation within nodes persistent	Seed-based	Horovitz et al. (2009)
			↓: Correlation PCC with MPFC became absent	
		25	↓: PCC, PHG, MPFC	ICA	Sämann et al. (2011)
	Light sedation	16	↑: PCC and areas outside of the DMN	Seed-based	Stamatakis et al. (2010)
		12	↓: General deceased connectivity, focal decreases PCC	ICA	Greicius et al. (2008)
	Anesthesia	20	↓: PCC/precuneus, MPFC, superior frontal sulci, parahippocampal gyrus, and bilateral TPJ	Seed-based and ICA	Boveroux et al. (2010)
		14	↑: PCC and STG	Seed-based	Martuzzi et al. (2010)
			↓: PCC and adjacent areas	
		18	↓: Reduction connectivity within the DMN and between the DMN and other networks	ICA	Schrouff et al. (2011)
	Hypnosis	18	↓ right middle and superior frontal gyrus	Seed-based	McGeown et al. (2009)
		12	↑: Middle frontal and bilateral angular gyri	ICA	Demertzi et al. (2011b)
			↓: PCC and bilateral parahippocampal areas	
	Comatose states	2	↓: Connectivity is absent in brain dead, decreased PCC, and thalamus connectivity	ICA	Boly et al. (2009)
			Preserved cortico-cortical connectivity	
		11	↓: Connections between PCC and MPFC	Seed-based and ICA	Soddu et al. (2012)
			Locked-in patients showed near to normal connectivity	
		14	↓: All areas showed less connectivity in disorders of consciousness, decrease of connectivity was negatively correlated with consciousness. PCC most significant decrease	ICA	Vanhaudenhuyse et al. (2010)
		13	Presence of DMN has prognostic value	ICA	Norton et al. (2012)
Executive control network	Light sleep	10	No difference	Seed-based	Larson-Prior et al. (2009)
	Slow wave sleep	25	Correlations within the network persist but decrease	ICA	Sämann et al. (2011)
	Light sedation	20	↓: Right: middle frontal and posterior parietal cortices.	Seed-based and ICA	Boveroux et al. (2010)
			Left: residual in middle frontal, PCC, and temporo-occipital cortices	
Salience	Slow wave sleep	14	↑: Connectivity between insula and left ACC	Seed-based	Martuzzi et al. (2010)
			↓: Decrease between connectivity in the insula and supplementary motor cortex and left middle frontal gyrus	
	Hypnosis	8	↑: Increases in mid-insula, primary sensory, and orbitofrontal cortex	Seed-based	Derbyshire et al. (2004)
Sensorymotor network	Light sleep	10	No difference	Seed-based	Larson-Prior et al. (2009)
	Slow wave sleep	14	↑: Connectivity within the network	Seed-based	Martuzzi et al. (2010)
	Light sedation	12	↑: Within-network increases	ICA	Greicius et al. (2008)
Auditory	Slow wave sleep	14	No difference	Seed-based	Martuzzi et al. (2010)
	Light sedation	20	No difference	Seed-based and ICA	Boveroux et al. (2010)
Visual	Light sleep	10	No difference	Seed-based	Larson-Prior et al. (2009)
	Light sedation	14	↑: Primary visual area with the cuneus and lingual gyrus	Seed-based	Martuzzi et al. (2010)
	Anesthesia	20	No difference	Seed-based and ICA	Boveroux et al. (2010)

*Upper arrow denotes increases in functional connectivity; lower arrow denotes decreases in functional connectivity. (DMN, default mode network; PCC, posterior cingulate cortex; PHG, parahippocampal gyrus; IPC, inferior parietal cortex; MPFC, medial prefrontal cortex; TPJ, temporoparietal junction; STG, superior temporal gyrus, ICA, independent component analysis)*.

Taken together, changes in the DMN functional connectivity in altered consciousness states could suggest modified self-related conscious mentation. Indeed, it has been suggested that in normal waking conditions, resting state activity in the posterior cingulate and frontal areas accounts for self-referential thoughts (Whitfield-Gabrieli et al., 2011). Therefore, it could be inferred that decreased connectivity in the DMN reflects restricted abilities for self-referential processing, like in patients with disorders of consciousness. One should keep in mind, though, that our limited understanding of the dynamic neural complexity underlying consciousness and its resistance to quantification in the absence of communication make it difficult to establish strong claims about self-consciousness in non-communicating subjects.

### DMN functional anticorrelations

Since the early studies of resting state, it was suggested that the brain’s baseline activity can be organized in two brain networks showing anticorrelated activity to each other: an “intrinsic” and an “extrinsic” network (Fox et al., 2005; Fransson, 2005; Golland et al., 2007; Tian et al., 2007). The “intrinsic” network coincides with the DMN and is involved in the same cognitive processes as the DMN. The “extrinsic” system encompasses lateral frontoparietal areas resembling the brain activations during goal-directed behavior and it has been linked to cognitive processes of external sensory input, such as somatosensory (e.g., Boly et al., 2007), visual (e.g., Dehaene and Changeux, 2005), and auditory (e.g., Brunetti et al., 2008). Previous studies showed that these two systems are of a competing character in the sense that they can disturb or even interrupt each other (e.g., Tian et al., 2007). Such anticorrelated pattern is also illustrated in activation studies on motor performance (Fox et al., 2007), perceptual discrimination (Sapir et al., 2005), attentional lapses (Weissman et al., 2006), and somatosensory perception of stimuli close to somatosensory threshold (Boly et al., 2007). We recently determined the cognitive-behavioral counterpart of such “resting state” activity and showed that activity in the DMN corresponded to behavioral reports of “internal” awareness (i.e., self-related thoughts). Conversely, subjective ratings for “external” awareness (i.e., perception of the environment through the senses) correlated with the activity of an “extrinsic” system (encompassing lateral frontoparietal cortices; Vanhaudenhuyse et al., 2011). These findings depict that the anticorrelated pattern between DMN and the extrinsic system is of functional significance to conscious cognition. With an aim to further characterize the role of these two systems to subjective awareness, we sought to modulate their relationship by means of hypnosis. We found that, as compared to a control condition of autobiographical mental imagery, there was a hypnosis-related reduction in connectivity in the “extrinsic” system, reflecting a decreased sensory or perceptual awareness. Interestingly, this modulated activity was paralleled to subjective reports of increased sense of dissociation from the environment and reduced intensity of “external thoughts” (Demertzi et al., 2011b).

**Figure 2:**
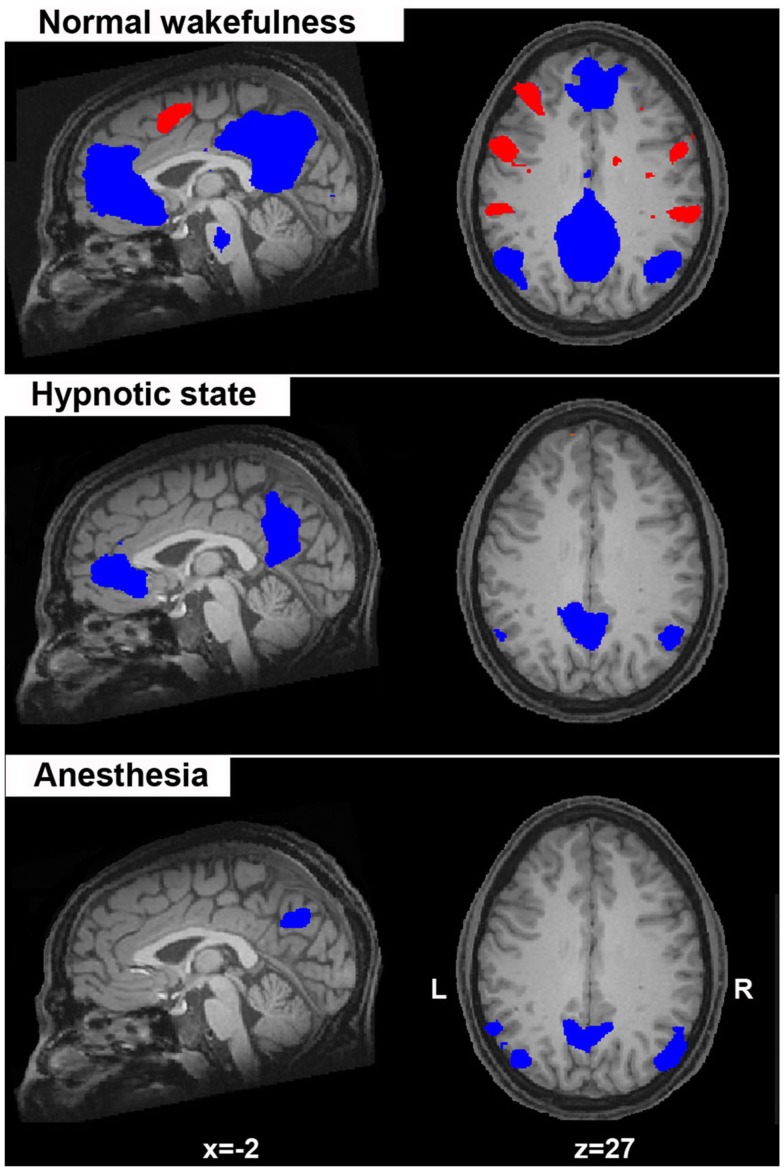
**Spontaneous fMRI BOLD activity in the default mode network (in blue; considered to reflect self-related mentation) anticorrelates with the activity of a lateral frontoparietal system (in red; considered to mediate conscious perception of the external world)**. Here, this anticorrelated activity is shown for normal wakefulness, hypnotic state, and during deep anesthesia. Of note is the absence of the activity in the “extrinsic” frontoparietal system in the two conditions of altered sense of awareness (hypnosis, anesthesia) which is considered as suggestive of a diminished “external” awareness (i.e., the perception of the environment through the senses). Statistical maps are thresholded at a false discovery error rate *p* < 0.05 and rendered on a structural T1 magnetic resonance image of a healthy subject (*x* and *z* values indicate Talairach coordinates of the represented sections).

Taken together these data indicate that DMN and anticorrelated extrinsic system activity underlies (at least partially) conscious ongoing mentation. It should be mentioned that fMRI anticorrelations were previously subject to debate in the literature. It has been argued, for instance, that fMRI functional anticorrelations are nothing more than noise in the signal due to regression of the brain’s global activity during data preprocessing (Anderson et al., 2010). Other data, however, suggest that the anticorrelations persist both with and without global signal regression, suggesting some underlying biological origins for this anticorrelated pattern (Fox et al., 2009; Chai et al., 2012). We would agree with the latter evidence which is supported by studies in unconscious conditions, such as anesthesia (Boveroux et al., 2010; Figure 2), sleep (Sämann et al., 2011), and in unresponsive patients (Boly et al., 2009) where these anticorrelations generally reduce or even disappear, accounting for their functional contribution to conscious cognition.

## Beyond the DMN: Resting State Activity in Multiple Cerebral Networks

Importantly, the different functions of a brain region cannot be understood in isolation, meaning in terms of functional segregation, but only in conjunction with regions it interacts with, that is in terms of functional integration (Seghier et al., 2010). Therefore, we further focus our review on other “large-scale higher-order” (bilateral executive control and salience networks) and sensorimotor-sensory (auditory, visual) RSNs, which can be consistently identified in healthy conditions (Figure 2).

The executive control network during normal wakefulness encompasses bilateral middle, inferior and superior frontal cortices, bilateral inferior parietal lobes, ACC/supplementary motor area (SMA), and bilateral insular cortices (Figure 1). Resting state independent components analysis identified this network in a lateralized manner. The left executive control network is thought to be more involved in cognitive and “language” paradigms while the right executive control network relates to perceptual, somesthetic, and nociception processing (Smith et al., 2009; Laird et al., 2011). Activity in both these two networks is reduced during deep sleep (Sämann et al., 2011) and anesthesia (Boveroux et al., 2010) whereas light sleep does not seem to mediate functional connectivity in these networks (Larson-Prior et al., 2009; Table 1). Taken together, these results highlight the involvement of the executive control networks in the perception of the external world, in line with previous suggestion that activity of these areas is a necessary condition for conscious (i.e., reportable) visual perception (Dehaene et al., 2003).

The salience network encompasses fronto-insular and ACCs (Figure 1) with connections to subcortical and limbic structures. In normal conditions, this network is implicated in the orientation toward salient emotional stimuli (Seeley et al., 2007), conflict monitoring, information integration, and response selection (Cole and Schneider, 2007; Roberts and Hall, 2008). It has been proposed that the salience network enables the switch between internal attention (the default mode) and task-related states (Menon and Uddin, 2010). The salience network has also been linked to pain-related processes both during acute stimulus-induced pain (Tracey and Mantyh, 2007), during resting state while anticipating pain (Ploner et al., 2010; Wiech et al., 2010), and after hypnotic suggestions for creating pain experiences in the absence of a noxious stimulus (Derbyshire et al., 2004). Under light sevoflurane sedation, increased connectivity between the ACC and the insula was observed, although connectivity between the insula and the secondary somatosensory cortex was reduced (Martuzzi et al., 2010). Analysis of the salience network in comatose states could be beneficial for the study of pain and possible suffering in these patients in the absence of external stimulation. Indeed, such stimulations are not always feasible due to sophisticated setups or due to patients’ clinical picture. Hence, salience network resting state analysis could shed light on the cerebral substrate that could account for patients’ orientation to salient stimuli, including painful ones.

The sensorimotor network resembles the activations seen in motor tasks (Biswal et al., 1995). In normal wakefulness it encompasses the SMA/midcingulate cortex, bilateral primary motor cortex, and bilateral middle frontal gyri (Biswal et al., 1995; Greicius et al., 2008; Figure 1). During light sedation the sensorimotor network shows increases in functional connectivity (Greicius et al., 2008; Martuzzi et al., 2010). To date, the above networks have not been further investigated under other unconscious states.

The auditory network, important in audition, such as tone/pitch discrimination, music, and speech (Laird et al., 2011) in normal wakefulness, encompasses primary and secondary auditory cortices, including Heschl’s gyrus, bilateral superior temporal gyri, and posterior insular cortex (Figure 1). During normal wakefulness, resting state independent component analysis (ICA) also identifies the visual network in three independent components (Figure 1). One network, the lateral visual network includes the middle temporal visual association area at the temporo-occipital junction and is most important in complex (emotional) stimuli (Laird et al., 2011). The other networks include medial and occipital visual networks, important in simple visual (e.g., a flickering checkerboard), and higher-order visual stimuli (e.g., orthography), respectively (Beckmann et al., 2005; Damoiseaux et al., 2006; Allen et al., 2011; Laird et al., 2011). No difference in connectivity was identified between both these primary auditory and visual sensory networks and light sleep (Larson-Prior et al., 2009), or between awake and sedation (Boveroux et al., 2010; Martuzzi et al., 2010). One study showed increased temporal synchrony in auditory and visual areas in light midazolam sedation (Kiviniemi et al., 2005). The visual cortex has been shown to possess higher amplitude of BOLD fluctuations when asleep (Fukunaga et al., 2006). This indicates that resting state activity continues in these areas during sleep, and thus transcends consciousness. Finally, reliably indicated as possessing functional connectivity is the cerebellum. This network is associated with action and somesthesis (Laird et al., 2011), but not yet thoroughly studied in altered states of consciousness.

## Analyzing Resting State Data from Pathological Brains: Methodological Issues

The clinical neuro-investigation of severely brain-injured patients with the resting state paradigm is technically easier compared to activation (Schiff et al., 2005) or “active” mental imagery protocols (e.g., Monti et al., 2010). This is because patients do not have to perform any task, and such data can have faster translation into clinical practice (Soddu et al., 2011). Depending on the adopted methodology, several issues need to be taken into account when analyzing resting state acquisitions from clinical populations. To date, two main approaches are employed; hypothesis-driven seed-voxel correlation analysis and data-driven ICA (see Table 1 for the adopted approach by each reviewed study). Each method has its own advantages, yet their methodological difficulties, especially in non-collaborative patients, which merit to be acknowledged.

### Hypothesis-driven method: Seed-based correlation analysis

The seed-voxel approach uses extracted BOLD time course from a region of interest and determines the temporal correlation between this signal (the seed) and the time course from all other brain voxels (Fox et al., 2005). This creates a whole-brain voxel-wise functional connectivity map of covariance with the seed region. It is the most straightforward method to analyze functional connectivity of a particular brain region. The method gives direct answers to specific hypotheses about functional connectivity of that region. It is attractive and elegant for many researchers as the data can be interpreted relatively easily when a well-defined seed area is used. When applying this approach to the study of resting state activity in patients with disorders of consciousness, several controversial issues arise. A first general issue concerns regressing out the global activity from the BOLD signal, which might induce spurious anticorrelations (Fox et al., 2009; Murphy et al., 2009). However, in the case of brain death (a condition where the brain totally lacks neuronal activity and arterial blood flow), this type of regression is an important step to obtain the obvious zero connectivity in this condition (Boly et al., 2009). Alternatively, a non-zero BOLD signal measured in brain death can be taken to be artifactual, contaminated by head motion or heart beating (Soddu et al., 2011). Next, patients with severe brain injuries may suffer from structural deformations resulting from traumatic brain injury and focal hemorrhages. Additionally, patients with severe chronic brain injuries usually develop atrophy and secondary hydrocephalus (i.e., *ex vacuo* dilation of the ventricles). This implies that even if a statistical structural normalization procedure has been performed, the selection of a proper seed region can become difficult and will require visual inspection by an expert eye. This issue adds to the already intrinsic challenges of an *priori* selection of the seed region which, in principle, can lead to as many possible overlapping networks as the number of possible seeds (Cole et al., 2010). Using seed-based analysis, other noisy confounds might be influencing the data (e.g., head motion, vascular activity, scanner artifacts). To reduce such noise, the BOLD signal can be preprocessed by regressing out head motion curves as well as ventricular and white matter signal, and each of their first-order derivative terms (Fox et al., 2005). Finally, as for all group-level analyses, one has to take into account the between-subject variability, such as cortical folding or functional localization between individuals or groups (Cole et al., 2010) which can be extremely challenging in severely deformed brains.

### Data-driven method: Independent component analysis

**Figure 3:**
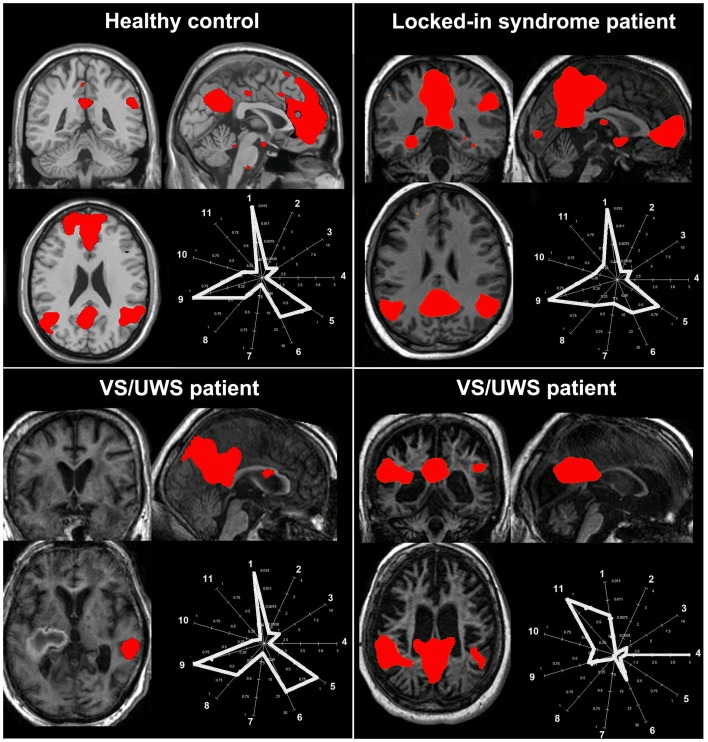
**The challenge of selecting the “right” independent component as the resting state network of interest in pathological conditions**. The figure illustrates the spatial pattern (brain maps, *z* values 0.8–10) and spatial-temporal properties (fingerprints: a representation of the component in a multidimensional space of parameters; De Martino et al., 2007) of the default mode network in healthy consciousness states (healthy subject, patient with locked-in syndrome; upper row) and in two patients with vegetative state/unresponsive wakefulness syndrome (VS/UWS; lower row). For the healthy control, the locked-in syndrome and the VS/UWS patient in the lower left corner, the default mode network shows the characteristic properties in both the spatial and the temporal domain (i.e., the fingerprints pick in the 0.02–0.05 Hz frequency band labeled with the number 9) even if for the VS/UWS patient the spatial pattern is only partially preserved. Of note is that the second VS/UWS patient exhibits the spatial pattern of the default mode network but importantly the time course of this component is characterized by high frequency fluctuations, in the 0.1–0.25 Hz frequency band and high spatial entropy (labeled, respectively, with the number 11 and 4 in the fingerprint). Therefore, such activity cannot be considered of neuronal origin. As a consequence, if the component selection was merely based on a spatial similarity test (e.g., with a predefined template), then this component could be erroneously selected and further statistically analyzed. A “compromise” in the selection of the appropriate network of interest in the space and time domain is needed to will eventually exclude non-neuronal contributions [Fingerprint labels: (1) degree of clustering, (2) skewness, (3) kurtosis, (4) spatial entropy, (5) autocorrelation, (6) temporal entropy, power: (7) 0–0.008 Hz, (8) 0.008–0.02 Hz, (9) 0.02–0.05 Hz, (10) 0.05–0.1 Hz, (11) 0.1–0.25 Hz].

Data-driven methods are used to analyze whole-brain connectivity patterns without the need of *a priori* seed regions. ICA is the most widely used methodology with high level of consistency in results within subjects (van den Heuvel and Hulshoff Pol, 2010). ICA divides an entire dataset into different maximally statistical independent components and thus is able to isolate cortical connectivity maps from non-neural signals (Beckmann et al., 2005). Spontaneous activity is therefore automatically separated from noise, such as head motion or physiological confounds (e.g., cardiac pulsation, respiratory, and slow changes in the depth and rate of breathing; Beckmann and Smith, 2004). This method has the advantage that it can evaluate and compare the coherence of activity in multiple distributed voxels (Cole et al., 2010). The advantage is that it divides different RSNs into different components. However, ICA does not provide any classification or ordering of the independent components. It is therefore perceived as more difficult to understand due to the complex representation of the data. The most straightforward method for labeling the components is by visual inspection, but this lacks reproducibility and could be hard to perform in cases with a large component dimensionality. Alternatively, an automatic selection is preferable but the way to choose the right independent component remains a delicate issue. By merely performing a spatial similarity test with a predefined template has been shown not to be successful for choosing the right component (Soddu et al., 2012). Some automatic approaches for component selection have been proposed, based on template matching using the “goodness of fit” as an outcome index. However, these methods have to be interpreted with care especially in cases of deformed brains as in patients with a traumatic brain injury or comatose state. It was recently proposed that when selecting independent components in patients populations, spatial, temporal, and a “compromise” between spatial and temporal properties of the network of interest need to be met (Soddu et al., 2012). For example, a component can be erroneously selected as the RSN of interest if the selection is based on the spatial pattern ignoring the properties in the time domain (Figure 3, bottom right panel). Additionally, the determination of the proper dimensionality (i.e., the “right” number of estimated components) remains unclear. Extracting many components can result in the spatial segregation of the network of interest into multiple sub-networks (Smith et al., 2009). It was shown, for example, that the use of 75 components can reduce the DMN into four components and the sensorimotor network in six (Allen et al., 2011). When applying ICA in pathological brains it is probably more useful not to select a large quantity of components, because high component dimensionality can further reduce the chances of identifying a network due to decrease in spatial pattern and spectral properties (Tohka et al., 2008).

Other techniques to analyze resting state data exist such as methods that focus on the (fractional) amplitude of low frequency fluctuations [(f)ALFF; Zang et al., 2007; Zuo et al., 2010), or on the small world characteristics using correlations and graph analysis (Bullmore and Sporns, 2009; Zalesky et al., 2012].

## Conclusions and Perspectives

The default mode network is the most widely studied network in the resting state literature and has been linked to self-related processes. To date, fMRI resting state studies show that DMN connectivity is reduced under altered states of consciousness, such as sleep, sedation/anesthesia, hypnotic state, and clinical states of disorders of consciousness (VS/UWS, MCS, coma, and brain death). Such connectivity alterations can be discussed in two non-mutually exclusive ways. On one hand, one can refer to these reductions in resting state connectivity during altered conscious states as reflecting reduced capacities for (conscious) cognitive processing (e.g., Vanhaudenhuyse et al., 2010). On the other hand, we can equally talk about persistent (albeit reduced) functional connectivity pattern in unconscious states, which transcends the level of consciousness, and which is considered as a physiologic baseline (e.g., Raichle et al., 2001). In any case, it seems that the purposes and questions of each study will eventually determine how such alterations can be further discussed and interpreted. Both the scientific and clinical implications for cognition seem to be the essence of resting state connectivity measurements.

At the scientific level, resting state analyses shed light on the necessary conditions needed for conscious awareness to take place. In other words, in the absence of external stimulation, resting state *functional* connectivity paradigms could quantify the minimal prerequisites under which cognitive processes can become “conscious.” This could mean that in the presence of an adequate neural substrate (i.e., the RSN), one could infer preserved capacities for conscious cognition. Of course the absence of functional connectivity cannot be taken as a proof for incapacity for conscious awareness. Indeed, it has been suggested that functional connections are best recruited after external stimulation (Honey et al., 2009). In any case, the sufficiency of the RSNs integrity to consciousness remains to be further determined with studies measuring *effective* connectivity (Churchland, 2007).

In summary, we here reviewed studies in resting state fMRI connectivity of “higher-order” associative cerebral networks (default mode, right and left executive control, and salience) and “lower-level” sensory (auditory and visual) and sensorimotor networks under various altered states of consciousness. As previously proposed, in order for humans to be conscious of something, incoming information (via sensory networks) needs to be made globally available to multiple brain systems via long-range neurons associative networks (Dehaene and Changeux, 2011). Here, the reviewed studies suggest that resting state connectivity is preserved but altered in most RSNs under physiological and pharmacological states, impeding information integration. It should be noted here that it was not among our aims to exhaustively review all spectrum of altered states of consciousness. Much research has been conducted in states of altered sense of awareness, such as in neuropsychiatric disorders (e.g., dementias and schizophrenia; for a review see Buckner et al., 2008), meditation (Brewer et al., 2011; Josipovic et al., 2012), and drug-related states such as alcohol (Esposito et al., 2010), amphetamine (Roberts and Garavan, 2010), or psychedelic drugs (Carhart-Harris et al., 2012) which in general show changes in the connection between the posterior cingulate and frontal areas. Resting state investigations have also been attempted using other modalities, such as electroencephalography (e.g., Lehembre et al., 2012). Rather, we here focused on RSNs obtained using fMRI. We reviewed changes in functional connectivity as a function of various states of wakefulness. This aim lies within our ultimate clinical goal to better document, manage and predict residual brain functioning of patients with disorders of consciousness. As these patients are incapable of functional communication with their environment, they might be wrongly diagnosed as unconscious when locked-in (Laureys et al., 2005) or when suffering from aphasia (Majerus et al., 2009). The ethical implications of erroneous diagnostics are apparent, especially when pain (Demertzi et al., 2009, 2012) and end-of-life issues (Demertzi et al., 2011a) are discussed.

At the clinical level, the study of resting state activity in pathological states of consciousness can become demanding due to both clinical and methodological issues. For example, patients who show increased prescan motion activity will need to be anesthetized to reduce the noise during data acquisition. Apart from the clinical issue of applying anesthetics to these vulnerable patients, the effect of anesthesia will need to be accounted for in the acquired data. This is added to the methodological challenge of the spatial normalization of severely deformed brains (Shen et al., 2007). Additionally, identified resting state connectivity patterns need to be interpreted according to the studied population. In brain death, for instance, it was shown that resting state fMRI activity is absent in line with the clinical neurological criteria for the diagnosis of death (Boly et al., 2009). Therefore, in cases where resting state activity, in the DMN for example, is identified, such findings can be pertained to motion and other artifacts, not indicative of neuronal activity (Soddu et al., 2011). The characterization of the fMRI functional connectivity of other RSNs in comatose states remains to be further elucidated. It can be expected, though, that in such severely constrained situations, like in disorders of consciousness, the functional integrity of most RSNs is considerably restricted accounting for patients’ limited capacities for conscious cognition.

Despite intrinsic limitations, resting state data are technically easier to obtain in patients’ population, as compared to auditory (Schiff et al., 2005) or visual (Monti et al., 2012) activation protocols or “active” mental imagery protocols (Monti et al., 2010; Bardin et al., 2011). The challenge now is twofold: first, to unravel the relationship (i.e., correlations, anticorrelations) between and among the RSNs under various conscious conditions. The second challenge is to move from static functional connectivity measurements to the assessment of the temporal dynamics of such associations, meaning looking at changes in functional connectivity across time. Such imperatives are justified when considering the nature of intrinsic brain activity, which is ongoing and which characterizes most areas of the brain, beyond the DMN (Raichle and Snyder, 2007).

## Conflict of Interest Statement

The authors declare that the research was conducted in the absence of any commercial or financial relationships that could be construed as a potential conflict of interest.
